# The Infusion of Piperacillin/Tazobactam with an Elastomeric Device: A Combined 24-H Stability Study and Drug Solution Flow Rate Analysis

**DOI:** 10.3390/ph17081085

**Published:** 2024-08-19

**Authors:** Laura Négrier, Anthony Martin Mena, Christian Dupont, Philémon Gamache, Jeanne-Olive Zimbril, Yasmine Abdoune, Youness Karrout, Pascal Odou, Stéphanie Genay, Bertrand Décaudin

**Affiliations:** 1 Univ. Lille, CHU Lille, ULR 7365—GRITA—Groupe de Recherche sur les formes Injectables et les Technologies Associées, F-59000 Lille, France; 2 Hôpital Universitaire Cochin, Assistance Publique—Hôpitaux de Paris, 75014, France—GIFAV—Groupe Interdisciplinaire Francophone sur les Accès Vasculaires, 75014 Paris, France; 3 Univ. Lille, INSERM, CHU Lille, U1008, F-59000 Lille, France

**Keywords:** piperacillin/tazobactam, flow rate, stability study, outpatient parenteral antimicrobial therapy (OPAT), continuous infusion, HPLC-UV

## Abstract

Bacterial respiratory tract infections (e.g., in patients with cystic fibrosis) may be treated with the intravenous infusion of a piperacillin/tazobactam (P/T) solution through an elastomeric device. In the present work, we combined a 24-h drug stability study with an assessment of the drug solution flow rate during an in vitro simulated infusion. Experiments were performed in triplicate with two excipient-free generic P/T solutions and an excipient-containing proprietary P/T solution in saline (all 50/6.25 mg/mL) released from an elastomeric infusion device at 32 °C. The P/T solutions’ stability was assessed by an HPLC-UV assay, pH and osmolality measurements, a visual assessment, and particle counting. Before these analyses, a forced degradation study was performed. To assess the flow rate, a precision scale was used to weigh the solution collected at the infusion line outlet. The stability criteria were <10% degradation and a flow rate within ± 15% of the nominal value over the 24-h infusion period: all three P/T solutions were found to be stable. The actual flow rate was lower than the expected flow rate; this difference was probably due to the drug solution’s high viscosity and must be taken into account in clinical use.

## 1. Introduction

Patients with bronchiectasis (whether related or not to cystic fibrosis) are prone to frequent bacterial bronchial infections, the treatment of which may require the intravenous infusion of antibiotics. Some patients may receive outpatient parenteral antimicrobial therapy (OPAT). Portable elastomeric devices are widely used for OPAT with prolonged or continuous infusion of antibiotics. According to clinical indications and stability data, the infusion time of injectable antibiotics can range from several hours to several days of infusion time [1,2]. Continuous infusion can optimize therapy by providing a plasma drug concentration four to eight times higher than the minimum inhibitory concentration for the pathogenic bacterium in question. The combination antibiotic piperacillin/tazobactam (P/T) is a key tool in the treatment of bronchial infections; it combines time-dependent efficiency and a broad spectrum of action. P/T is supplied as a powder for reconstitution and dilution to give a solution for infusion [3]. P is a ureidopenicillin, and T is a ß-lactamase inhibitor [3]. P/T is effective against infections by multidrug-resistant, Gram-negative bacteria (such as *Pseudomonas aeruginosa*), which have become increasingly frequent in recent years. Prolonged intravenous (IV) treatment with P/T is particularly useful for combating these multidrug-resistant, Gram-negative strains. The study described below was carried out in collaboration with pulmonologists who prescribe and follow up OPAT on a regular basis. The drug is usually administered every 12 h at a dose level of 12/1.5 g per day. Stability data for longer infusion times are not available, or data available do not specify whether a forced degradation study has been performed [4,5]. However, 24-h infusions would facilitate the treatment for the patient, the nursing staff, and the healthcare system by (i) decreasing the number of interventions required, (ii) reducing the amount of waste produced, and (iii) increasing the cost-effectiveness of care. Home treatment is often preferred by the patient; it improves quality of life and also reduces the risk of cross-infection.

Sterile, single-use, non-programmable elastomeric devices are now routinely used for the continuous parenteral/IV administration of drugs [6]. They often take the form of a rigid, transparent plastic shell, together with a silicone or polyisoprene elastomer reservoir containing the drug [6]. The drug is dispensed by mechanical pressure as a function of the reservoir’s elastic properties, according to Poiseuille’s law [7,8]. The choice of the elastomeric diffuser will depend on the target infusion volume, rate, and duration. Several factors can affect the infusion flow rate: the type of elastomer, various physicochemical factors (e.g., the solution’s viscosity and the ambient temperature), the height difference between the reservoir and the flow regulator, and the position of the flow regulator on the patient’s skin (ensuring maintenance a few degrees below body temperature, i.e., the temperature for which the elastomeric device is calibrated). Each manufacturer decides on their elastomeric device’s exact calibration temperature and the diluent used. For the infusion of dextrose 5%, for example, the Folfusor^®^ LV10 (Baxter) is calibrated at 31.1 °C, and the Accufuser^®^ (Woo Young Medica) is calibrated at 32 ± 2 °C [9,10]. Furthermore, the clinical guide from Vygon states that temperatures of 31.1 and 33.3 °C are achieved when the Luer Lock Connector is taped to a peripheral skin site and a central skin site, respectively. It is well known in the literature that the skin surface temperature depends on the region of the body studied [11]. Given that an elastomeric device is calibrated for operation at a fixed temperature, these findings clearly indicate that the choice of the venous access site (peripheral versus central) will influence the flow rate of a drug infused with an elastomeric device.

In order to guarantee therapeutic effectiveness and the patient’s safety, two major issues need to be addressed by diffuser users: the drug solution’s physicochemical stability, and flow rates potentially below the nominal value as a result of changes in viscosity and temperature [12,13]. Firstly, the stability over time of antibiotics infused using portable diffusers is still a major concern [14]. Chemical instability produces a risk of treatment failure. Degradation of the active ingredient may jeopardize effectiveness and/or safety (as observed for toxic derivatives of ceftazidime, for example) [15]. A recent literature review highlights the lack of stability data for certain antibiotics and the need for an international consensus on the stability variables to be studied for portable dispensers [16]. One of the main limitations of in vitro studies is that they take little account of the patient’s exact living conditions. Many factors can influence drug stability: exposure to light, the materials used in the diffuser, the pH, concentration and composition of the solution for infusion, the storage conditions, the prior or extemporaneous preparation of the diffuser, and the infusion rate and duration [2,17]. Temperature is one of the major factors mentioned in the literature. Most stability studies (including those performed by device manufacturers) are carried out at 25 or 37 °C [18,19,20]. In real life, however, the diffuser is placed on the patient’s skin; depending on the patient’s level of physical activity and exposure or not to the sun, the temperature of the antibiotic solution may rise to well over 25 °C. This temperature difference can affect the diffuser’s delivery rate. Most studies look at physicochemical stability only and fail to consider the reliability of the actual infusion rate [14,19]. Given that elastomeric devices are not linked to an electronic monitor, there are no alarms to be triggered in the event of a device malfunction or incorrect positioning on the skin. The success of OPAT therefore also depends on good patient education and appropriate training for healthcare professionals [8,17].

In the present work, we combined a 24-h stability study with an assessment of the actual infusion rate of a 50/6.25 mg/mL P/T saline solution delivered with a FOLFUSOR LV10^®^ device at 32 °C (31.90 ± 0.58 °C) and at a nominal rate of 10 mL/h. By combining these two aspects, our objective was to determine the potential effectiveness and safety of this drug infusion, i.e., whether infusion with an elastomeric device complied with the prescribed target dose. Lastly, we compared two generics (from Fresenius Kabi and Mylan) with the proprietary drug (Tazocilline^®^, from Pfizer).

## 2. Results

### 2.1. Validation Variables for the HPLC-UV Method

The variances were homogeneous, and the analysis of variance (ANOVA) confirmed that the linear model was applicable. Linearity was confirmed at a 95% confidence level for piperacillin (from 150 to 350 µg/mL) and tazobactam (from 18.75 to 43.75 µg/mL) (Table 1 and Table S1). The R^2^ values were always > 0.99, whatever the drug supplier. The results of the forced degradation study demonstrated that the method was stability-indicating.

### 2.2. The Forced Degradation Study

No degradation products were co-eluted with P, T, or the excipients. It was necessary to heat the solution for 1 h at 75 °C to degrade piperacillin and for 5 h at 75 °C to degrade tazobactam. One hour in 1 N HCl at room temperature was enough to degrade tazobactam, while 20 min with 0.03 N HCl at room temperature was enough to degrade piperacillin. Five minutes with 0.001 N NaOH at room temperature was sufficient to degrade piperacillin, whereas 5 min with 0.01 N NaOH at room temperature were required for tazobactam. In 0.9% H_2_O_2_ at room temperature, incubations of 12 and 90 min deteriorated piperacillin and tazobactam, respectively (see Figure S1). Very similar results were found for the proprietary drug (Figure S2). Under all conditions, tazobactam appeared to be more stable than piperacillin.

### 2.3. The Stability Study

Before infusion, the solutions were clear and colorless but the number of subvisible particles exceeded the European Pharmacopoeia (EP) acceptance limits. During infusion, however, the number of subvisible particles fell below the EP acceptance limits. Under all conditions, the P/T concentrations remained between 90 and 110% of the initial (T0 h) concentration (Table 2, Table 3 and Table 4). All three solutions tested (the proprietary drug and the two generics) gave similar chromatograms (Figure S3). The pH and osmolality varied by less than 0.5 pH units and 10 mOsm/kg, respectively. These results showed that the drugs were chemically stable over the 24-h infusion period, under all conditions.

### 2.4. Drug Solution Flow Rates and Viscosity

**Figure 1:**
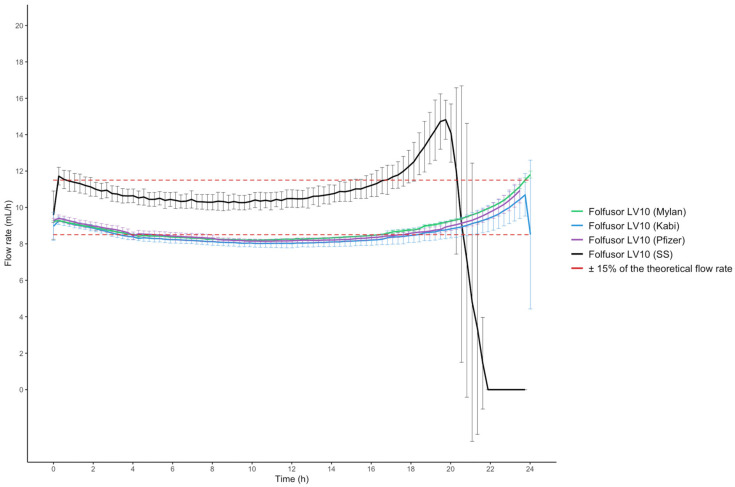
The solution flow rates in the four conditions (n = 3 per condition).

The flow rate was higher when the elastomeric device was filled with physiological saline solution (SS) only, relative to P/T solutions. The solution densities were approximately 1.027 for the excipient-free generics, 1.030 for the proprietary drug (Pfizer, with excipients), and 1.000 for SS. The control infusion with SS was complete (i.e., the elastomer device had emptied itself) in less than 24 h. With the Folfusor^®^ LV10, the infusion rate was the same for all three P/T solutions (Figure 1).

**Figure 2:**
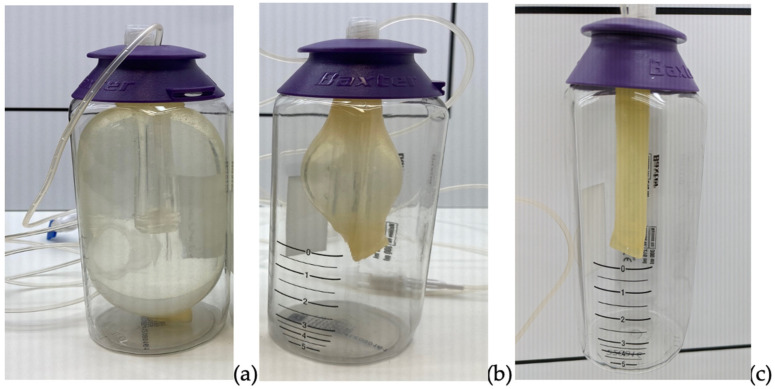
Illustrative photos of elastomeric devices: (**a**) a device before the 24-h infusion, (**b**) a P/T-loaded device after the 24-h infusion, and (**c**) an SS-loaded device after the 24-h infusion.

The elastomeric devices’ visual aspect was assessed (using a black and white panel) before and after the 24-h infusion (Figure 2). After the infusion, the elastomeric devices still contained some P/T solution, and SS-loaded devices (controls) were empty. The proportions of the initial volume remaining in the elastomeric device after the 24-h infusion (8.12 ± 1.46%, 8.23 ± 1.86%, and 10.04 ± 2.03% for the Mylan generic P/T, the Fresenius Kabi generic P/T and the Pfizer proprietary P/T, respectively) were consistent with the calculated values (Table 5).

The three P/T solutions appeared to be slightly more viscous (as measured with a Brookfield viscometer) than water for injection (WFI), and all the viscosity values were higher at 22 °C than at 32 °C (n = 3) (Table S2).

## 3. Discussion

Before conducting the present study, we reviewed the literature on the stability of P/T obtained from different suppliers. The literature studies were carried out at various temperatures (15–25, 35, and 37 °C), with various diluents (5% dextrose, saline solution, citrate Buffer, or WFI), with or without protection from light, and at various concentrations (9/1.15–90/11.25 mg/mL) [1,5,8,14,16,21]. However, the study of chemical stability did not always involve forced degradation study, and the physical stability was not always coupled to the study of chemical stability. For example, Loeuille et al. decided to work at body temperature (37 °C) and with a higher drug concentration (66.7 mg/mL P/8.3 mg/mL T). The researchers found that the P/T solution was unstable in the Baxter Folfusor^®^ LV10, and suggested that this was due to a pH variation of more than 0.5 units over the 24-h study period—even though the dose level was maintained at over 90% of the initial value [2]. In contrast, we did not observe a pH variation of more than 0.5 units; this interstudy disparity might be due to the higher concentration and higher temperature studied by Loeuille et al. which emphasizes the importance of checking all the stability variables. Jamieson et al. reconstituted and administered drug solutions using the Folfusor^®^ LV10 and the B.Braun Medical Easypump II; the P/T concentrations were 80/10 and 22/3 mg/mL, respectively. The results indicated that the P/T combination prepared in SS did not have the required stability when it was stored for seven days in the refrigerator and then for 24 h at 32 °C. Along with a short communication by El Saghir et al., Jamieson et al.’s study was the only one to show that buffering the P/T solution stabilized the drugs [1,22]. However, the buffer used by Jamieson et al. (0.3% *w/v* citrate-buffered saline pH7) is not marketed in all countries. Most stability studies described in the literature used generic P/T preparations. We reasoned that it would be interesting to compare solutions from several suppliers, especially since some contained excipients and others did not. Hence, to the best of our knowledge, the present study is the first to have compared the stability of P/T solutions for injection prepared with drugs from three different manufacturers. Our results showed that it is always difficult to choose the temperature for a stability study with portable dispensers because (i) the device is close to the body, and (ii) the body temperature can vary easily [23]. It is, therefore, necessary to educate the patient on the correct use of elastomeric devices. For instance, the patient should avoid putting a duvet on the elastomeric device and should not take a hot shower. Furthermore, the requirements for stability testing in studies involving body temperatures set out in the UK National Health Service Yellow Covered Document changed between 2015 (the third edition) and 2019 (the fifth edition) [24,25]. The third edition specified that a fixed temperature of 37 ± 2 °C should be used for “in-use near-to-body studies”. However, in the fifth edition, the temperature of 32 ± 1 °C is recommended for in-use near-to-body studies with elastomeric devices [24,25]. 

Another constraint linked to the use of an elastomeric device is that the flow rate is influenced by the viscosity of the drug solution [13], the type of diluent, and the temperature. Although a margin of error (±15% of the theoretical flow rate) is acceptable [6], differences in nursing practice may increase the likelihood of underdosing. For example, a nurse might disconnect an elastomeric device that is not completely empty after a 24-h infusion. The lack of drug delivery might then lead to treatment failure, with various consequences: the development of bacterial resistance, hospital admission, or prolongation of a hospital stay. The need to deliver the full dose has already been highlighted in several studies [26,27,28]. The calculated flow rates for drug solutions appeared to be just above the −15% limit vs. the theoretical flow rate [6]. Perks et al. stated that 18 g/240 mL of P/T diluted in SS and infused with the Folfusor^®^ LV10 at 31.1 °C did not meet the minimum criteria for infusion of 90% of the total volume over a 24-h period: 34.08 mL (14.20% of the initial total volume) was not delivered [27]. At the concentration used in our study (50/6.25 g/240 mL), 12.31 mL (5.08% of the initial total volume) was not delivered [27]. Hence, our results are similar to those reported by Perks et al. Furthermore, a short communication by Quintens et al. mentioned that for a concentration of 16 g/264 mL of P/T infused at 33 °C, the residual volume after 24 h of infusion corresponded to 19% of the daily dose. Quintens et al.’s results emphasize the significant impact of the solution’s viscosity. According to Quintens et al.’s graphs, the viscosity was about 0.95 mPa.s (1 Cp = mPa.s) for a 60.6 mg/mL piperacillin solution at 33 °C and 0.925 mPa.s for a 50 mg/mL piperacillin solution. Another graph indicated that the flow rate was 9 mL/h with a viscosity of 0.95 mPa.s and 9.25 mL/h with 0.925 mPa.s, rather than the theoretical value of 10 mL/h with the Infusor^®^ LV10 [13]. The viscosity values observed in our study are in line with those reported by Quintens et al. Our values obtained at 22 °C were higher than those obtained at 32 °C, which can be explained by the Hagen-Poiseuille law.

To the best of our knowledge, Quintens et al.’s study is the only one in the literature to have compared the proprietary P/T with an excipient-free, generic preparation; the drug solution flow rates were the same. Our results demonstrated the stability of the P/T solution over the 24-h infusion period. Compliance with the EP recommendations for the number of subvisible particles can be explained by the presence of a particulate filter in the Folfusor^®^ LV10, which removed the high number of subvisible particles before the solution was placed in the elastomeric device.

Our results also emphasized the relatively low flow rates obtained for infusions of P/T solutions. This can be explained by the high viscosity of the P/T solution, which depends on the temperature and the drug concentration [13,27,29]. In the Hagen-Poiseuille law, the flow rate is inversely proportional to the viscosity. The effects of the temperature and viscosity of an injectable solution on the drug solution flow rate had previously been reported for 5-fluorouracil [29]. It is also important to note that (i) the skin temperature may vary according to the thickness of the clothing worn, and (ii) the drug solution flow rate may vary according to both the air temperature and the skin temperature [12,30]. Unfortunately, these variables are difficult to fix under in vitro conditions. Our results also showed that (as also mentioned in the manufacturer’s specifications) the flow rate was not constant over the 24-h infusion. It is known that the flow rate increases initially, then decreases slowly, stabilizes, and then increases markedly over the last few hours of infusion. During the first few minutes of the infusion, the P/T solution prepared at room temperature gradually warms up in the incubator; this might explain the increase in the drug solution flow rate. Two other variables might impact the flow rate: the type of diluent and the position of the restrictor relative to the Folfusor^®^ LV10 [27]. The restrictor must be positioned at the same height as the elastomeric chamber. Hence, we used a transparent medical incubator (i.e., the type used for premature infants) so that the flow restrictor’s position could be monitored. Another advantage of using a medical incubator is that it avoids pinching the elastomeric device’s infusion line. To the best of our knowledge, this is the first study to have used a medical incubator to assess the infusion flow rate of an elastomeric device. Regarding the choice of the diluent, the Folfusor^®^ LV10 is calibrated with dextrose 5% at 31.1 °C. The manufacturer specifies that the use of SS increases the flow rate by 10%, relative to dextrose 5%. This factor, together with our slightly higher temperature (31.90 ± 0.58 °C), might explain why the flow rate was in the upper range of the authorized ± 15% (Figure 2). Nevertheless, our decision to use SS (rather than dextrose 5%) was based on its greater availability in the pharmacy and more frequent use in OPAT. However, it would have been interesting to set up a comparative control infusion with a dextrose 5% solution (as in the manufacturer’s calibration). We did not observe marked differences in flow rate between generic solutions and the proprietary solution. This might be explained by the fact that the excipients accounted for approximately 3% of the weight of the Tazocilline^®^ P/T powder [27]. We studied the Folfusor^®^ LV10, which is made of polyisoprene and is resistant to alcohols, acids, bases, and polar solvents. Furthermore, the Folfusor^®^ LV10 is suitable for use in low-temperature environments, so preparations can be stored in a cool place. It would have been interesting to compare the Folfusor^®^ LV10 with a silicone elastomeric device. 

Our study had several limitations. Firstly, it would have been interesting to perform more experiments and thus increase the study’s statistical power. Secondly, and as in many of the literature studies, we did not record stability data after 24 h of infusion. Thirdly, we could have studied the physicochemical stability and flow rate until the elastomeric device was completely empty. Hence, the study’s design could have been improved by extending the study duration accordingly. In addition, this work could also be completed by a container-content interaction study and a drug compatibility study if the P/T solution was infused simultaneously with another solution. Nevertheless, our study paves the way for a new method for assessing the stability of injectable drugs infused with elastomeric devices. To facilitate the interpretation of the results, our model could be adapted and standardized for the assessment of elastomeric devices in general. We consider that physicochemical stability and the drug solution flow rate should always be studied together, in order to conclude with confidence that the infusion is effective and safe.

In view of our results, we have several recommendations for OPAT nurses. For example, we paid attention to the displacement volume of the P/T powder in contact with SS (0.7 mL per g P/T, according to some summaries of product characteristics). For example, 4 g/0.5 g P/T powder for a solution for infusion displaces 3.15 mL [31]. The reconstitution solution must be drawn up with the syringe, and the volume must then be adjusted to the desired amount. After the 24-h infusion, the nurse could empty the residual volume in the elastomeric device into a glass; this would make it possible to estimate the P/T dose not administered to the patient and provide feedback to the medical team. The monitoring would be more accurate, and the patient could be told to check his/her infusion on a regular basis. The study by Cassettari et al. also demonstrated the value of (i) an antimicrobial stewardship program for OPAT and (ii) the importance of a prescription assessment by infectious disease specialists (58% of the prescriptions were modified after this assessment [32]). It might be interesting to study the role of the elastomeric device’s filling volume on variations in the flow rate infusion for a given concentration. The clinical relevance of reducing the infusion volume and increasing the drug concentration should be studied, in order to provide clinicians with better guidance. Given that a nurse is not always available at home, it would be valuable to involve patients in the care process and train them in the correct use of elastomeric devices. Infusion protocols should be standardized, in order to limit the risk of further variations in drug solution flow rates and decrease the variability in clinical practices observed at present.

## 4. Materials and Methods

### 4.1. Chemicals, Reagents and Diluents

Potassium dihydrogen phosphate for analysis, orthophosphoric acid 85%, and acetonitrile for HPLC were respectively purchased from Supelco (EMSURE^®^ ISO, 1.04873.1000, Merck, Darmstadt, Germany), VWR chemicals prolabo (UN1824, GPR rectapur, Fontenay-sous-Bois, France), and Honeywell (UN1648, 34851-2.5 L, Riedel-de-Haën, Offenbach, Germany). Proprietary tazocillin 4.5 g vials came from Pfizer (batch FR21009, 11/2024, Paris, France), and the two generic P/T 4.5 g powders for injection came from Mylan (batch 2150917FR, 06/2024, Lyon, France) and Fresenius Kabi (batch 18Y0758, 10/2025, Labesfal, Portugal). The generics did not contain excipients, whereas the proprietary drug was formulated with citric acid and EDTA [33]. For the forced degradation study, HCl 37% was obtained from Supelco (Merck, Darmstadt, Germany), NaOH 30% was obtained from VWR Chemicals Prolabo (28217.292, 1 L, Fontenay-sous-Bois, France), and H_2_O_2_ 3% was purchased from Gilbert Laboratories (120 mL, Batch G240910, 01/2027, Hérouville Saint-Clair, France). Ultrapure water was obtained from a Purelab^®^ classic water system (ELGA LabWater, Antony, France). Baxter (Guyancourt, France) provided the SS 0.9% (Viaflo 250 mL bag, Batch 23H18E4G, 07/2025) and the WFI (Viaflo 500 mL bag, Batch 22001E3C, 02/2025). All materials were used as received.

### 4.2. Preparation of Solutions and the In Vitro Infusion Set-Up

As mentioned in the European summary of product characteristics [34], the antibiotic P/T 4.5 g consists of a powder that can be reconstituted with WFI or SS in a volume of 20 mL. This initial solution can then be diluted with SS to obtain a solution for infusion. In accordance with standard drug preparation guidelines, three vials of P/T 4.5 g powder were reconstituted in 20 mL of WFI. A syringe (50 mL BD Luer-Lock, 300865, batch 2112, 07/2026) screwed onto an 18-gauge needle (SOL-M, K111815, batch 08109008, 09/2026) was used to collect the 20 mL from each vial of P/T 4.5 g. This volume was then topped up with SS to 50 mL *qs*. The three 50-mL syringes were pooled and mixed with 90 mL of SS in an empty infusion bag (SLB Medical, Genas, France, 250-mL phthalate-free, PB02502852T20CPP149, batch 412306, 10/2028)) to obtain a homogeneous solution with a final volume of 240 mL. This 50/6.25 mg/mL P/T solution was then transferred into the FOLFUSOR^®^ LV10 (Baxter, 2C4063K, Batch 23A026, 01/01/2026). As mentioned above, the FOLFUSOR LV10^®^ diffuser is made of polyisoprene; this synthetic polymer has much the same properties as natural latex but lacks the allergen. Folfusor^®^ LV10 includes a particulate matter filter on the fluid path of the solution. Its pore size is less than or equal to 5 µm. The filling volume of 240 mL was between the minimum (216 mL) and maximum (300 mL) volumes recommended by the manufacturer. The same reconstitution and dilution protocol was used for all three drug preparations, i.e., the two generics (P/T Mylan and Fresenius Kabi) and the proprietary drug (Tazocilline^®^, Pfizer).

**Figure 3:**
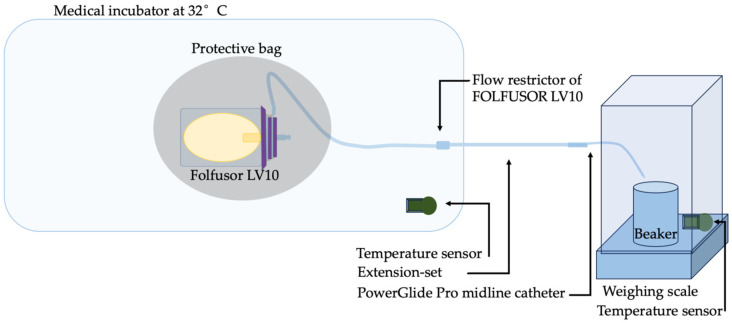
The in vitro infusion setup.

Our in vitro procedure was designed to mimic the real infusion conditions in an outpatient setting or at home as closely as possible. A Folfusor^®^ LV10 portable diffuser was wrapped in an opaque bag (to protect it from light) and placed in a medical incubator (Médipréma MP5, ISIS Intelligent System for Incubator Surveillance, France) thermostated at 32 °C. We chose to study the Folfusor^®^ LV10 at a temperature of 32 °C; this value lies between 31.1 °C (corresponding to the skin surface temperature associated with peripheral venous access and also that used by the manufacturer for device calibration) and 33.3 °C (corresponding to the skin surface temperature associated with central venous access). The flow restrictor was located in the incubator at the same height as the diffuser (as recommended by the manufacturer). The diffuser tubing was connected to a polyethylene extension set (length: 50 cm; diameter: 1 mm; 1155.05, Vygon, Ecouen, France) and a PowerGlide Pro midline catheter (BARD, 6F120080, Salt Lake City, UT, USA) leading into an empty beaker placed and tared on a Sartorius weighing scale at room temperature (AX423, d: 0.001 g; maximum: 420 g, Dislab, Lens, France). The scale was operated with a custom macro developed in-house. The addition of the extension set and the midline catheter faithfully reproduced the home use of the infusion setup. To ensure that the temperature readings were reliable, the medical incubator was turned on several hours before the start of the in vitro infusion. The temperatures inside and outside the incubator were recorded every 10 min, using temperature sensors linked to ThermoTracer^®^ software (version 3, Oceasoft, Montpellier, France). One sensor was placed on the scale (to check the room temperature; measured value: 22.25 ± 0.73 °C), and the second was placed in the incubator set to 32 °C (measured value: 31.90 ± 0.58 °C (Figure 3). The humidity level could be checked once, using a small Digital Hygrometer (ThermoPro, Newark, DR, USA) in the medical incubator and the weighing scale. The medical incubator showed a humidity level of 50% and a weighing scale of 55%.

Four preparations were investigated: the generic P/T from Mylan, the generic P/T from Fresenius Kabi, the proprietary P/T from Pfizer, and SS only (the control). Each solution was tested in triplicate.

### 4.3. The 24-H Stability Study

**Figure 4:**
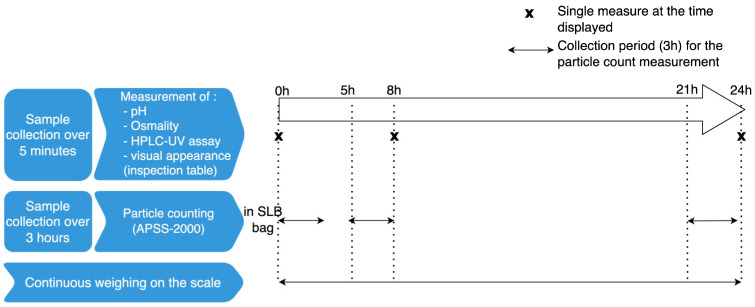
The sample measurement times during the 24-h in vitro infusion.

To determine the physicochemical stability of the drug solutions over 24 h, samples were collected in a beaker at the outlet of the infusion line and then analyzed using an HPLC-UV assay, pH and osmolality measurements, a visual assessment, and particle counting. All variables were studied in triplicate. For the HPLC-UV assays, each sample from each triplicate experiment was diluted in three replicates. A new empty beaker was tared for each new sample. The weight was recorded every 10 s during the 24-h infusion. To determine the infusion flow rate, the scale measured the weight of the collected solution every 10 s from T0 h to T24 h of infusion, i.e., from the beginning to the end of the theoretical 24-h infusion. The various variables were measured at various time points during the procedure (Figure 4). 

In order to accurately determine the solution flow rate, each solution’s density was established beforehand so that the weights of the drug solutions could be converted into volumes. A Coriolis mass flowmeter (Mini cori-flow, Bronkorst, Montigny-lès-Cormeilles, France) and FlowSuite software (v.1.0.10, Bronkorst) were used for this purpose. To check the consistency of the calculated flow rates, the elastomeric devices (empty and filled) were weighed at T0 h (the empty diffuser before preparation and the filled diffuser after preparation) and T24 h.

#### 4.3.1. Chromatographic Apparatus and Conditions

The HPLC-UV system (Shimadzu, Noisiel, France) was equipped with a DGU-20A 5R degassing unit, one Prominence LC-20AD quaternary pump, a Prominence SIL-20AC automatic injector, a CTO-10AC oven, and a SPD-M20A diode array detector (Nexera X2). The system was operated with LabSolutions^®^ CBM-20A software (v.5.57, Shimadzu). The HPLC-UV analysis lasted 25 min; it featured a 5 µL sample injection volume and a mobile phase flow rate of 1.2 mL/min. The oven temperature was set to 25 °C, and the autosampler temperature was set to 5 °C. Preliminary assays demonstrated the stability of samples at 5 °C, as also reported by other researchers [2]. In line with the literature methods, the UV detection wavelength was set to 280 nm for piperacillin and 210 nm for tazobactam [1,2].

**Figure 5:**
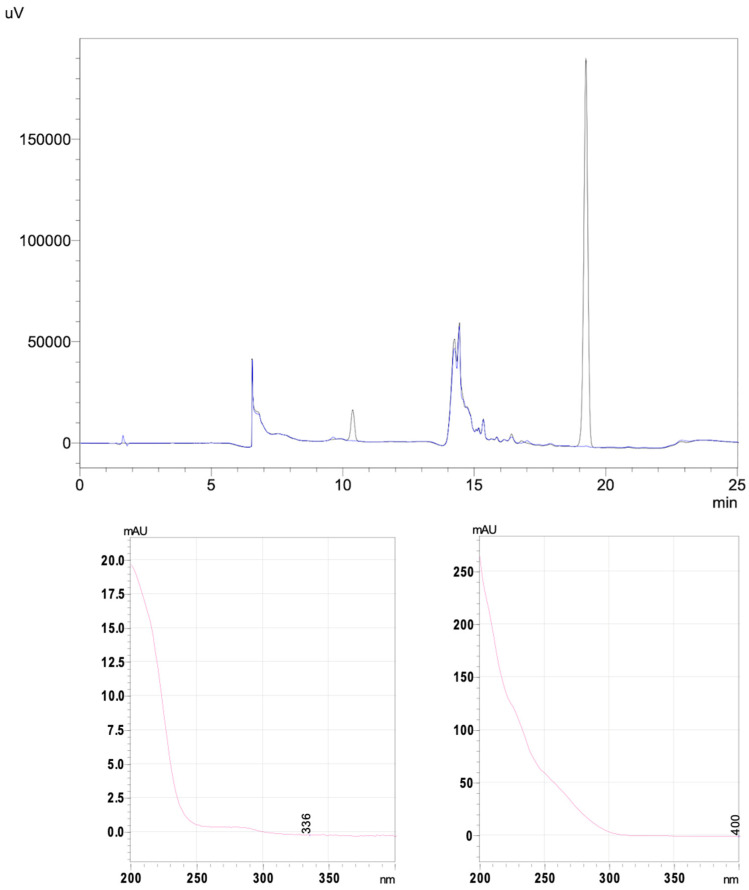
Chromatogram (in black: P/T solution from Fresenius Kabi, at point of 0 h of infusion overlapped with a chromatogram from mobile phase in blue) and UV spectra (measurement wavelength: 210 nm) for the P/T(Fresenius Kabi, T0 h) HPLC-UV assay (retention times: 10.5 min for tazobactam and 19.3 min for piperacillin).

Our choice of the mobile phase drew on the results of several previous studies [1,2]. It consisted of an aqueous phase (phosphate buffer 0.02 M, adjusted to pH 2.5 with orthophosphoric acid 85%) and an organic phase (acetonitrile). The pH of the phosphate buffer was measured and adjusted using a pH meter (HI 5221, Hanna Instruments, Lingolsheim, France) and an InLab^®^ Micro pH electrode (Mettler Toledo, Greifensee, Switzerland). The buffer was degassed with an ultrasonic cleaner and then filtered using a vacuum pump filtration system and a membrane filter (GH Polypro 47 mm and 0.45 µm hydrophilic polypropylene, Pall Life Sciences). The stationary phase consisted of a Gemini^®^ 150 × 4.6 mm × 5 µm, 110 Å C18 column (Phenomenex, Le Pecq, France) and its pre-column (AJ0-87680, Phenomenex). Gradient elution mode was used, and the organic phase proportion ranged from 0 to 30% (Table 6 and Figure 5).

After the sample had been collected at the outlet of the infusion line, it was diluted twice with ultrapure water and then filtered (regenerated cellulose filter, cut-off 0.2 µm, Chromoptic, Courtaboeuf, France).

#### 4.3.2. The HPLC-UV Forced Degradation Study

To ensure that the HPLC-UV stability method was stability-indicating, we conducted forced degradation assays. Degradation of 10 to 25% was required, in order to determine whether primary degradation products or impurities were co-eluted with the P/T and (for Tazocilline^®^) the excipients. Four degradation conditions were tested individually, with exposures to heat (in a drying oven), acid, base, and an oxidant. The stability studies were performed in accordance with the guidelines issued by the French Society of Clinical Pharmacy (Société Française de Pharmacie Clinique) and the Evaluation and Research Group on Protection in Controlled Atmospheres (Groupe d’Evaluation et de Recherche sur la Protection en Atmosphère Contrôlée) [35]. Initial tests were carried out on solutions of piperacillin sodium, tazobactam sodium, and the P/T diluted in SS and then with WFI. 250 µL of a 1000/125 µg/mL P/T stock solution was placed in contact with 250 µL of the degradant for the desired length of time at room temperature (except for the heat exposure experiment). This solution was then mixed with 250 µL of neutralizing solution at the same concentration as the degradant (i.e., HCl, if the degradant was NaOH) or ultrapure water (if the neutralizing solution was not necessary), and then 250 µL of ultrapure water. This three-fold dilution was chosen so that in the absence of degradation, the final P/T concentration would be 250 µg/mL/31.25 µg/mL. Each sample was analyzed using HPLC-UV immediately after the last dilution. The forced degradation experiments involved HCl 0.03 M and 1 N, NaOH 0.01 and 0.001 N, 0.9% H_2_O_2_, and a drying oven at 75 °C.

#### 4.3.3. Validation of the HPLC-UV Stability-Indicating Method

The stability-indicating method was performed in accordance with the 1992 guidelines issued by the French Society of Pharmaceutical Science and Technology (Société Française des Sciences et Techniques Pharmaceutiques) [36]. The assays were carried out in three series of five concentrations, over three days. The stock solutions were prepared at the nominal concentration indicated for the diffuser and were then diluted with ultrapure water. On the chromatograms, the retention times for tazobactam and piperacillin were 10.5 min and 19.3 min, respectively. The homogeneity of the variances was assessed with a Cochran test (*p* < 0.005). To ensure that the linear model was appropriate, an analysis of variance (ANOVA) was applied. The mean recovery and the regression line were then determined.

#### 4.3.4. Measurements of pH and Osmolality

The pH was measured with a potentiometer, in accordance with the EP 2.2.3 [37]. In the absence of a pH acceptance criterion in the literature, we deemed that a maximum variation of ±0.5 pH units (relative to the value at T0 h) was acceptable. Osmolality was measured by measuring the solutions’ freezing point depression, in accordance with the EP 2.2.35 [38]. In the absence of an osmolality acceptance criterion in the literature, we deemed that a maximum variation of ±10 mOsmol/kg (relative to the value at T0 h) was acceptable. Solutions for injection should be isotonic; for example, the Infusion Standards of Practice states that solutions of more than 600 mOsmol/L should not be administered by the peripheral venous route [39]. Hence, we checked that the solutions’ osmolality did not exceed 600 mOsmol/kg. Experiments were performed in triplicate (n = 3). 

#### 4.3.5. Visual Assessment

The macroscopic aspect of the antibiotic solutions was checked to ensure that the solutions were colorless and free of cloudiness. The container and contents were inspected visually for the presence of particles visible to the naked eye, using an inspection table (Mirage V1.1D, Sterigene, Franconville, France). The EP 2.9.20 recommends monitoring the particle count in injectable solutions in their infusion containers, using a black and white panel and a lamp holder [40]. It further states that particulate contamination of injectable solutions may come from a variety of sources and should be reduced as much as possible. Solutions must be clear. The diffusers were inspected, but because they were opaque, we also inspected the solution in the collection beakers.

#### 4.3.6. Particle Counts

To determine whether the infusion solutions were contaminated with particles not visible to the naked eye, we applied the EP light-blocking method. For containers with a nominal capacity above 100 mL, the preparation passed the test if the following conditions were met: the mean count should not exceed 25 per mL for particles 10 µm or more in size and 3 per mL for particles 25 µm or more in size. The particles were counted using an APSS-2000^®^ machine and SamplerSight Pharma software (version 3.0 SP2 3.0.2.15744, both from Particle Measuring Systems, Boulder, CO, USA). Four measurements of 5 mL samples were taken. The first measurement was systematically discarded, as recommended by the EP [41].

### 4.4. Viscosity Measurements

A DV-1 Brookfield viscometer (DV1MRVTJ0, Brookfield Engineering Laboratories, Inc., Stoughton, MA, USA) was used to measure the viscosity of WFI (the reference solution), and each of the three 50/6.25 mg/mL P/T solutions in SS (the generics from Fresenius Kabi and Mylan Laboratories, and the proprietary drug from Pfizer laboratory). The spindle was an RV2, and the spindle rotation speed was set to 100 rpm. All solutions were maintained at 22 or 32 °C in a water bath (WNB 7, Memmert, Schwabach, Germany) and measured in triplicate. The temperature probe supplied with the DV-1 Brookfield viscometer was immersed in the water bath. After a few minutes, the viscometer readings were multiplied by 0.1 for our spindle/speed combination.

### 4.5. Expression of the Results

Quantitative variables were presented as the mean ± standard deviation. For HPLC-UV assays, the 95% confidence interval was calculated. Chemical stability was defined as a concentration that remained within 90–110% of the initial value (including the 95% confidence interval) throughout the 24-h infusion period. The drug concentration at the outlet of the infusion line at each time point was expressed as a proportion (in %) of the initial (T0 h) concentration. The flow rate was plotted as the mean trend over time, and the results were formatted using RStudio software (version 2022.02.0, R Foundation for Statistical Computing, Vienna, Austria). The measured flow rate had to be within ±15% of the nominal flow rate noted on the elastomeric device.

## 5. Conclusions

Our present results demonstrated the value of assessing both a drug solution’s physicochemical stability and its flow rate out of an elastomeric device. Thus, we found that a 50/6.25 mg/mL P/T solution remained physicochemically stable (90–110% of the T0 h concentration) during a 24-h infusion with a Folfusor^®^ LV10 at 32 °C but was not fully infused; some of the solutions remained in the elastomeric infusion device. These results are valid for the proprietary drug (Tazocilline^®^, from Pfizer) as well as the two generics studied (from Fresenius Kabi and Mylan). The percentage of the volume of piperacillin solution not administered to the patient was evaluated between 8 and 10%. According to the data, filtration of the P/T solution is necessary for any type of elastomeric device. In stability studies involving elastomeric devices, we consider that the physicochemical stability and drug solution flow rate should be evaluated systematically; this would provide clinicians with the information needed to ensure the safe management of their patients. Our results also show that pharmacists, clinicians, and nurses should be made more aware of the properties of elastomeric devices and should be trained in their correct use. Lastly, the present work might lead to the development of a potentially standardizable method for the evaluation of elastomeric devices.

## Figures and Tables

**Table 1:** Validation criteria for the analytical HPLC-UV method.

Validation Variables		Piperacillin (Mylan, Fresenius Kabi)	Tazobactam (Mylan, Fresenius Kabi)	Piperacillin (Pfizer)	Tazobactam (Pfizer)
Calibration range		150–350 µg/mL	18.75–43.75 µg/mL	150–350 µg/mL	18.75–43.75 µg/mL
Determination coefficient		0.997	0.999	0.999	0.996
Regression coefficients	Slope	867.85	4897.28	842.07	4763.45
	Intercept	7535.48	1247.50	3264.58	3560.96
Limit of detection (µg/mL)		8.227	0.576	4.488	1.279
Limit of quantification (µg/mL)		16.454	1.152	8.976	2.558
Cochran’s test	Experimental value	0.3966	0.334	0.4067	0.3155
	Theoretical value (α = 5%; 5; 8)	0.4564	0.4564	0.4564	0.4564
ANOVA (non-linearity)	Experimental value (α = 5%; 1; 3)	13,310.91	43,118.28	43,334.05	8284.46
	Theoretical value	4.11	4.11	4.11	4.11

**Table 2:** Mean ± standard deviation (95% confidence interval) P concentration (%), T concentration (%), pH, osmolality, particle count ≥ 10 µm, and particle count ≥ 25 µm for the Fresenius Kabi generic (n = 3).

Generic P/T (Fresenius Kabi)	T0 h (Bag Preparation)	T0 h	T8 h	T24 h
Appearance of the solution	Clear and colorless	Clear and colorless	Clear and colorless	Clear and colorless
P concentration%	/	100.0 ± 2.27	99.08 ± 0.64	97.91 ± 1.44
T concentration%	/	100.0 ± 9.62	98.64 ± 7.79	97.39 ± 6.13
pH	5.4 ± 0.0	5.3 ± 0.1	5.1 ± 0.1	5.0 ± 0.0
Osmolality (mOsm/kg)	417 ± 4	417 ± 4	415 ± 3	418 ± 2
Particles ≥ 10 µm/mL	396 ± 103	5 ± 4	2 ± 1	5 ± 4
Particles ≥ 25 µm/mL	3 ± 2	0 ± 0	0 ± 1	0 ± 0

**Table 3:** Mean ± standard deviation (95% confidence interval) P concentration (%), T concentration (%), pH, osmolality, particle count ≥ 10 µm, and particle count ≥ 25 µm for the Mylan generic (n = 3).

Generic P/T (Mylan)	T0 h (Bag Preparation)	T0 h	T8 h	T24 h
Appearance of the solution	Clear and colorless	Clear and colorless	Clear and colorless	Clear and colorless
P concentration%	/	100.0 ± 1.17	98.36 ± 0.70	95.40 ± 1.16
T concentration%	/	100.0 ± 1.47	98.76 ± 0.80	99.88 ± 1.94
pH	5.4 ± 0.0	5.4 ± 0.0	5.2 ± 0.1	4.9 ± 0.0
Osmolality (mOsm/kg)	414 ± 3	415 ± 2	416 ± 3	415 ± 1
Particles ≥ 10 (µm/mL)	31 ± 12	3 ± 3	2 ± 2	6 ± 3
Particles ≥ 25 (µm/mL)	1 ± 1	0 ± 1	0 ± 0	1 ± 1

**Table 4:** Mean ± standard deviation (95% confidence interval) P concentration (%), T concentration (%), pH, osmolality, particle count ≥ 10 µm, and particle count ≥ 25 µm for the Pfizer proprietary drug (n = 3).

Proprietary Drug (Pfizer)	T0 h (Bag Preparation)	T0 h	T8 h	T24 h
Appearance of the solution	Clear and colorless	Clear and colorless	Clear and colorless	Clear and colorless
P concentration%	/	100.0 ± 1.04	97.06 ± 1.18	96.04 ± 0.87
T concentration%	/	100.0 ± 3.45	103.01 ± 0.68	99.38 ± 0.16
pH	6.1 ± 0.0	6.1 ± 0.0	6.0 ± 0.0	5.8 ± 0.0
Osmolality (mOsm/kg)	439 ± 3	442 ± 1	442 ± 4	441 ± 4
Particles ≥ 10 (µm/mL)	90 ± 8	7 ± 5	3 ± 1	5 ± 1
Particles ≥ 25 (µm/mL)	1 ± 1	1 ± 1	0 ± 0	0 ± 1

**Table 5:** Mean ± standard deviation weights and volumes of the Folfusor^®^ LV10 and the infusion solutions (Mylan, Fresenius Kabi, and Pfizer) before and after the 24-h infusion (n = 3).

	Generic P/T (Mylan) in the Elastomeric Device	Generic P/T (Fresenius Kabi) in the Elastomeric Device	Proprietary Drug (Pfizer) in the Elastomeric Device
Weight of the empty Folfusor^®^ LV10 (g)	62.04 ± 0.35	61.94 ± 0.30	61.76 ± 0.73
Weight of the filled Folfusor^®^ LV10, before infusion (g)	307.58 ± 0.61	305.30 ± 0.78	308.27 ± 1.28
Calculated weight of solution before infusion (g)	245.55 ± 0.95	243.36 ± 0.48	246.52 ± 1.72
Calculated volume of solution before infusion (mL)	239.09 ± 0.92	236.96 ± 0.46	239.34 ± 1.67
Calculated weight of solution after the 24-h infusion (g)	20.02 ± 3.60	20.29 ± 4.59	24.84 ± 5.01
Calculated volume of solution after the 24-h infusion (mL)	19.50 ± 3.50	19.76 ± 5.47	24.12 ± 4.86
Proportion of infusion solution not infused (volume, %)	8.12 ± 1.46	8.23 ± 1.86	10.04 ± 2.03

**Table 6:** The gradient elution mode in the HPLC-UV method.

Analysis Time (min)	Organic Phase/Aqueous Phase %
0–3	0/100
3.5–11	8/92
11.5–20	30/70
20.5–25	0/100

## Data Availability

Data is contained within the article and Supplementary Material.

## Supplementary Materials

The following supporting information can be downloaded at: https://www.mdpi.com/article/10.3390/ph17081085/s1, Figure S1: Chromatograms from P/T solution (Fresenius Kabi) (measurement wavelength: 210 nm) of breakdown after forced degradations.; Figure S2: Chromatograms for the proprietary drug (Tazocilline®, Pfizer) (measurement wavelength: 210 nm) of breakdown after forced degradations; Figure S3: Chromatograms showing the HPLC-UV assays carried out at the time point of 0h (black) and 24h (blue) of the stability study.; Table S1: Relative bias and accuracy for the analytical HPLC-UV method; Table S2: Mean ± standard deviation viscosities of the P/T solutions and water for injection (WFI) at 22 °C (the room temperature) and 32 °C (the incubator temperature) (n = 3).
