# Supplementary material for: The Infusion of Piperacillin/Tazobactam with an Elastomeric Device: A Combined 24-H Stability Study and Drug Solution Flow Rate Analysis
Source: Pharmaceuticals (Basel). 2024 Aug 19;17(8):1085. doi: 10.3390/ph17081085 (PMC11360378; doi:10.3390/ph17081085)
Supplement: Supplementary file 1 [file pharmaceuticals-17-01085-s001.zip › pharmaceuticals-3120234-supplementary.pdf]

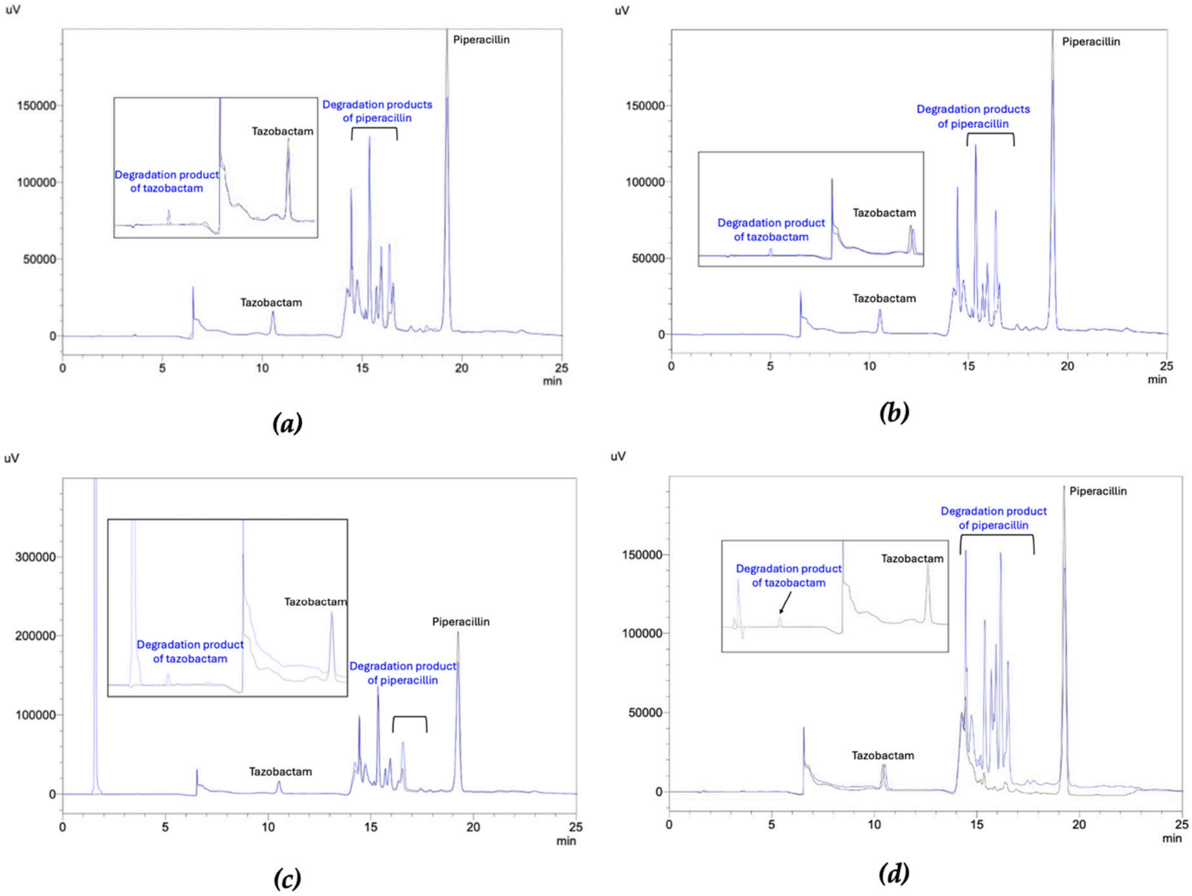

Figure S1: Chromatograms from P/T solution (Fresenius Kabi) (measurement wavelength: 210 nm) of breakdown after forced degradations (T0h without degradation : black and after forced degradation: blue) (retention times: 10.5 min for tazobactam and 19.3 min for piperacillin) (a) degradation with heating (75°C for 1 h for piperacillin, and 75°C for 5 h for tazobactam); (b) degradation with NaOH 0.001 N for 5 min for piperacillin and with NaOH 0.01 N for 5 min for tazobactam; (c) degradation with H_2O_2 0.9% 12 and 90 min; (d) degradation with HCl 0.03 N for 20 min for piperacillin and HCl 1 N for 1h for tazobactam. The main chromatogram shows the degradation of piperacillin, and the inset shows the degradation of tazobactam.

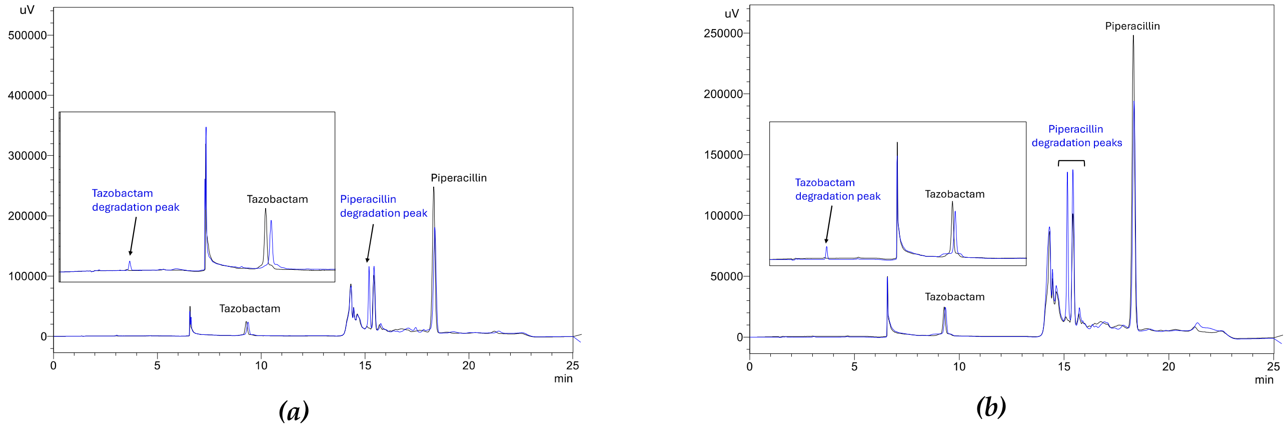

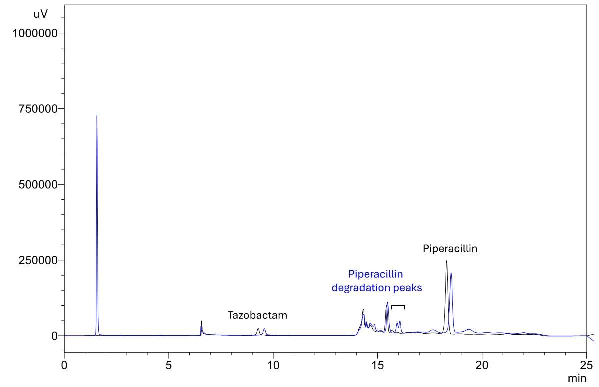

(c)

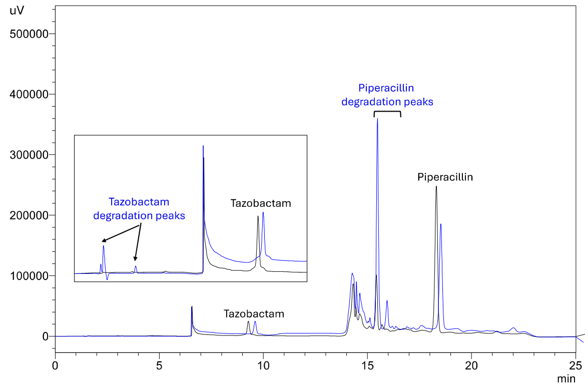

(d)

Figure S2: Chromatograms for the proprietary drug (Tazocilline®, Pfizer) (measurement wavelength: 210 nm) of breakdown after forced degradations (T0h without degradation : black and after forced degradation: blue) (retention times: 10.5 min for tazobactam and 19.3 min for piperacillin) (a) degradation with heating (75°C for 1 h for piperacillin, and 75°C for 5 h for tazobactam); (b) degradation with NaOH 0.001 N for 5 min for piperacillin and with NaOH 0.01 N for 5 min for tazobactam; (c) degradation with H_2O_2 0.9% 90 min; (d) degradation with HCl 0.03 N for 40 min for piperacillin and HCl 1 N for 1h for tazobactam. The main chromatogram shows the degradation of piperacillin, and the inset shows the degradation of tazobactam.

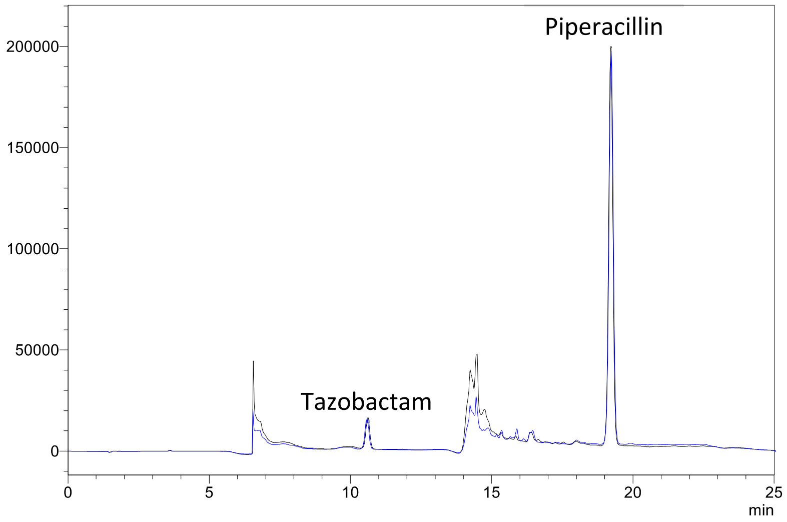

(a)

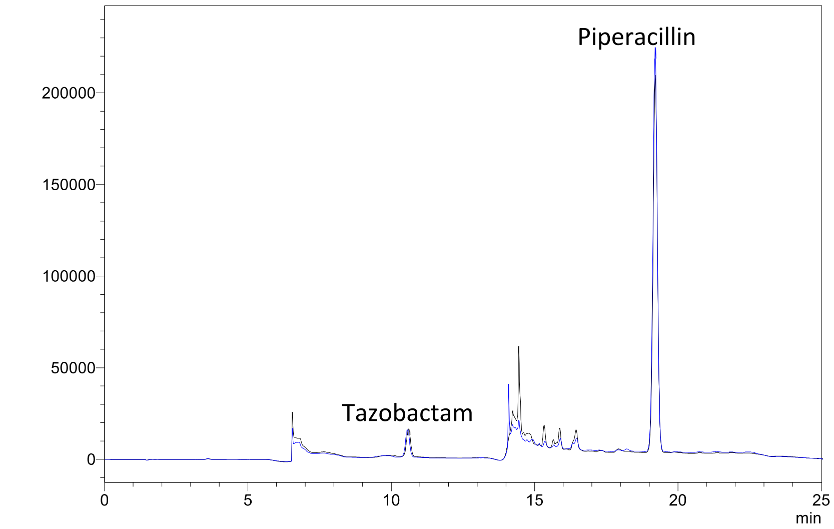

(b)

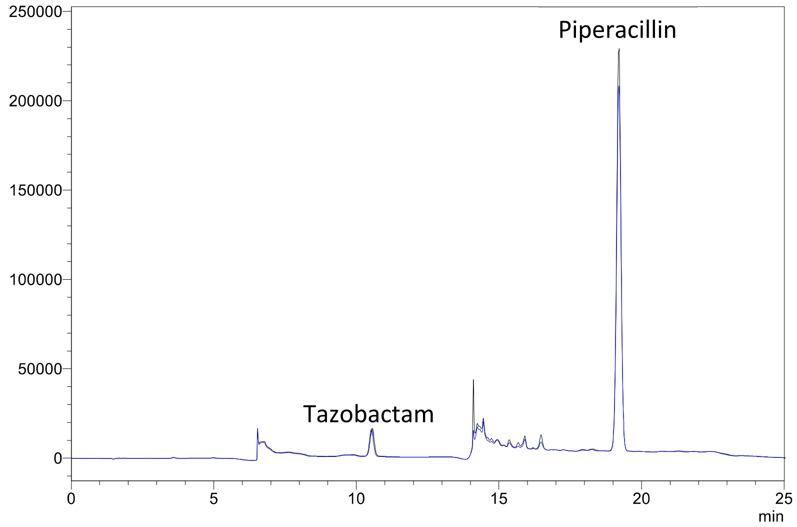

(c)

Figure S3: Chromatograms showing the HPLC-UV assays carried out at the time point of 0h (black) and 24h (blue) of the stability study and overlapped for : (a): P/T solution from Fresenius Kabi (generic), (b): P/T solution from Mylan (generic) and (c): Tazocilline® solution from Pfizer (proprietary drug).

Table S1: Relative bias and accuracy for the analytical HPLC-UV method.

	Concentration (µg/mL)	Relative bias (%)	Recovery factor (%)
Piperacillin (Mylan, Fresenius Kabi)	150	-0.7	99.3
	200	0.61	100.61
	250	0.18	100.18
	300	-0.12	99.88
	350	-0.08	99.92
Tazobactam (Mylan, Fresenius Kabi)	18.75	-0.2	99.79
	25	0.42	100.4
	31.25	-0.17	99.84
	37.5	-0.15	99.84
	43.75	0.1	100.09
Piperacillin (Pfizer)	150	-0.07	99.93
	200	0.06	100.06
	250	0.09	100.09

	300	-0.13	99.87
	350	0.04	100.04
Tazobactam (Pfizer)	18.75	-0.17	99.84
	25	0.01	100
	31.25	0.12	100.13
	37.5	0.13	100.13
	43.75	-0.13	99.86

Table S2: Mean ± standard deviation viscosities of the P/T solutions and water for injection (WFI) at 22°C (the room temperature) and 32°C (the incubator temperature) (n=3).

		**Generic P/T (Mylan)**	**Generic P/T (Fresenius Kabi)**	**Proprietary drug (Pfizer)**	**WFI (reference solution)**
22°C	Mean viscosity ± standard deviation (Cp)	1.15 ± 0.05	1.13 ± 0.02	1.12 ± 0.00	1.00± 0.00
	Exact measuring temperature (°C)	21.97 ± 0.25	22.10 ± 0.00	22.13 ± 0.15	22.00 ± 0.00
	% torque	2.88 ± 0.12	2.83 ± 0.06	2.77 ± 0.06	2.60 ± 0.00
32°C	Mean viscosity ± standard deviation (Cp)	1.0 ± 0.04	1.03 ± 0.02	0.97 ± 0.02	0.92 ± 0.00
	Exact measuring temperature (°C)	32.03 ± 0.06	31.50 ± 0.36	31.90 ± 0.10	31.80 ± 0.00
	% torque	2.53 ± 0.10	2.57 ± 0.06	2.43 ± 0.06	2.30 ± 0.00
